# TLR9 Mediated Tumor-Stroma Interactions in Human Papilloma Virus (HPV)-Positive Head and Neck Squamous Cell Carcinoma Up-Regulate PD-L1 and PD-L2

**DOI:** 10.3389/fimmu.2019.01644

**Published:** 2019-07-16

**Authors:** Paramita Baruah, Jessica Bullenkamp, Philip O. G. Wilson, Michael Lee, Juan Carlos Kaski, Ingrid E. Dumitriu

**Affiliations:** ^1^Department of Ears, Nose and Throat (ENT), St. George's University Hospitals NHS Foundation Trust, London, United Kingdom; ^2^Molecular and Clinical Sciences Research Institute and Cardiology Clinical Academic Group, St. George's, University of London, London, United Kingdom; ^3^Department of Pathology, St. George's University Hospitals NHS Foundation Trust, London, United Kingdom

**Keywords:** fibroblasts, HNSCC, HPV, PD-1, PD-L1, PD-L2, TLR9

## Abstract

**Background:** The co-inhibitory receptor PD-1 is expressed in many tumors including head and neck squamous cell carcinoma (HNSCC) and is an important immunotherapy target. However, the role of PD-1 ligands, PD-L1, and particularly PD-L2, in the tumor-stromal cell interactions that cause a tumor-permissive environment in HNSCC is not completely understood and is the focus of our study.

**Methods:** Expression of PD-L1 and PD-L2 was analyzed by immunohistochemistry *in situ* in HNSCC tumor tissue. Co-cultures were established between stromal cells (fibroblasts and macrophages) and human papilloma virus (HPV)-positive and HPV-negative HNSCC cell lines (HNSCCs) and PD-1 ligands expression was analyzed using flow cytometry.

**Results:** PD-L1 and PD-L2 were expressed both in tumor cells and stroma in HNSCC tissue *in situ*. *In vitro*, basal expression of PD-L1 and PD-L2 was low in HNSCCs and high on fibroblasts and macrophages. Interestingly, HPV-positive but not HPV-negative HNSCCs increased the expression of both PD-1 ligands on fibroblasts upon co-culture. This effect was not observed with macrophages. Conversely, both fibroblasts and macrophages increased PD-1 ligands on HPV-positive HNSCCs, whilst this was not observed in HPV-negative HNSCCs. Crucially, we demonstrate that up-regulation of PD-L1 and PD-L2 on fibroblasts by HPV-positive HNSCCs is mediated via TLR9.

**Conclusions:** This work demonstrates in an *in vitro* model that HPV-positive HNSCCs regulate PD-L1/2 expression on fibroblasts via TLR9. This may open novel avenues to modulate immune checkpoint regulator PD-1 and its ligands by targeting TLR9.

## Introduction

Advanced stage head and neck squamous cell carcinoma (HNSCC) is treated with a combination of surgery, radiotherapy, and chemotherapy with considerable morbidity and mortality. A significant advance in knowledge was the discovery that the oncogenic human papilloma virus (HPV, subtypes 16 and 18) associates with 60–70% of oro-pharyngeal cancer, a common HNSCC (1). Additionally, the observation that HPV-positive HNSCC patients have a better prognosis than those with HPV-negative HNSCC raises interesting questions about the immune response to the HPV-antigen in HPV-positive HNSCC.

Circulating T cells specific for HPV with anti-tumor activity have been identified in patients with HNSCC by us and others (2, 3), indicating an immune response to HPV virus. However, HNSCC develops and progresses in spite of this because tumor antigen-specific T cells fail to eliminate the tumor. We have previously shown that circulating HPV-antigen specific CD8^+^ T cells in patients with p16-positive HNSCC express high levels of the co-inhibitory receptor programmed cell death-1 (PD-1) (3), which may compromise their cytotoxic function (4). PD-1 is expressed by activated T cells following antigen encounter and it limits the proliferation and cytokine production of effector T cells (5, 6). The inhibitory actions of PD-1 are triggered by interaction with its ligands, PD-1 ligand 1 (PD-L1, B7-H1, or CD274), and PD-L2 (B7-DC or CD273). These ligands compete for binding to PD-1 and their expression pattern is complex. PD-L1 is constitutively expressed by immune cells (T cells, B cells, dendritic cells, macrophages) and also by non-haematopoietic parenchymal cells in many tissues (7). In contrast, PD-L2 expression is reportedly restricted to antigen presenting cells (dendritic cells, macrophages, and B cells) (7).

PD-L1 is also expressed in many tumors (8–12), and more recently tumor PD-L2 expression has been described (13). Some of the mechanisms proposed to drive expression of PD-L1 in tumors include interferon-γ (IFN-γ) produced by tumor infiltrating lymphocytes, genomic alterations, and activation of oncogenic signaling pathways (14–20). However, additional mechanisms are likely to exist. PD-1/PD-L1 axis is a major immune checkpoint important in the prognosis of several solid tumors (melanoma, hepatocellular carcinoma, and more recently HNSCC) (21–24) and is being clinically targeted for immunotherapy (25–29). Recent work on antibodies targeting PD-1 in advanced melanoma has yielded encouraging improvement in survival (28) and clinical trials are currently investigating the efficacy of anti-PD-1 and anti-PD-L1 monoclonal antibodies in recurrent and/or metastatic HNSCC (30–32).

Stromal cells such as tumor-associated macrophages and fibroblasts have important roles in generating an immunosuppressive tumor milieu (33). The role of PD-L1 and in particular PD-L2 in the tumor-permissive function of stromal cells in HPV-positive and HPV-negative HNSCC is yet to be completely understood. In this study we investigate whether interactions between tumor and stromal cells influence the expression of PD-1 ligands in HNSCC. We demonstrate that PD-L1 and PD-L2 are expressed in both tumor and stromal cells in HNSCC tissue from treatment-naïve patients. We present novel data that HPV-positive but not HPV-negative HNSCC cell lines (HNSCCs) increase PD-L1 and PD-L2 expression on fibroblasts. Of note, we demonstrate that the up-regulation of PD-L1 and PD-L2 on fibroblasts driven by HPV-positive HNSCCs is mediated via TLR9 as it was abrogated by the TLR9-specific antagonist ODN TTAGGG. Interestingly, chloroquine—an endosomal TLR inhibitor—selectively abrogated PD-L2 up-regulation. This is the first report demonstrating TLR9 involvement in regulation of PD-L1 and PD-L2 expression in the interaction between HPV-positive HNSCCs and stromal cells. Our findings have potential implications for PD-1/PD-L1/PD-L2 immune checkpoint modulation in HPV-positive HNSCC by unveiling TLR9 as an alternative target.

## Materials and Methods

### HNSCC Tissue Samples

Immunohistochemistry was performed on tumor biopsies from nine treatment-naive HNSCC patients with newly diagnosed cancers. Patients' characteristics are summarized in Table 1. To reduce the impact of inflammatory comorbidities on the expression of PD-1 and its ligands, strict exclusion criteria were applied and patients with co-existing inflammatory disorders such as autoimmune diseases, diabetes, renal failure, and cardiac disease were excluded from the study. The study was carried out in accordance with the recommendations of the Research Ethics Committee London-Chelsea who approved the study; all study subjects gave written informed consent in accordance with the Declaration of Helsinki.

**Table 1:** Demographic characteristics of head and neck cancer patients.

**Patient**	**Age**	**Gender**	**Tumor site**	**TNM**	**p16**
P1	61	F	Tonsil	T1N2bM0	Positive
P2	72	M	Tonsil/BOT	T4N2bM0	Positive
P3	62	M	Tonsil	T1N2aM0	Positive
P4	60	M	Tonsil	T3N0M0	Positive
P5	50	M	PFS/PCA	T4N0M0	Positive
P6	49	F	Uvula	T1N0M0	Negative
P7	83	M	PFS/PCA	T4N0M0	Negative
P8	50	F	BOT	T4N2cM0	Negative
P9	60	M	Tonsil/BOT	T4N2bM0	Negative

*BOT, base of tongue; PCA, postcricoid area; PFS, pyriform sinus*.

### Cell Lines

The human HNSCC lines UPCI-SCC-099 (SCC099) and UPCI-SCC-154 (SCC154) were purchased from DSMZ (Braunschweig, Germany). The SCC099 line was HPV-negative while the SCC154 line was HPV-positive. HNSCCs were maintained in Dulbecco's modified Eagle medium (DMEM, Sigma, #D6546) supplemented with 100 U/ml penicillin, 100 μg/ml streptomycin, 15 mM L-glutamine, 1 × non-essential amino acids and 10% heat-inactivated fetal calf serum (FCS, Corning) (culture medium). Primary human BJ fibroblasts (ATCC CRL-2522) were cultured in culture medium without non-essential amino acids.

### Primary Human Monocyte-Derived Macrophage Differentiation

Peripheral blood mononuclear cells (PBMCs) were isolated from healthy blood donors by density gradient centrifugation using Histopaque (Sigma-Aldrich; #10771). Monocytes were isolated by adherence to tissue culture-treated 12-well-plates by plating 3–5 × 10^6^ PBMCs per well in Iscove's modified Dulbecco's medium (IMDM, Sigma-Aldrich; #I3390) supplemented with 100 U/ml penicillin, 100 μg/ml streptomycin, 15 mM L-glutamine and 5% heat-inactivated pooled human serum (Lonza; #14-490E). Following incubation for 1 h at 37°C non-adhering cells were removed and culture medium was replaced. The culture medium was exchanged after 3–4 days and monocytes were differentiated into macrophages by 6–7 days culture at 37°C. Macrophage differentiation was routinely verified by staining with monoclonal antibodies against CD14 (APC; BD Biosciences; #555399) and CD68 (FITC; #562117; BD Biosciences).

### Co-cultures

1 × 10^5^ BJ fibroblasts were co-cultured with SCC099 or SCC154 cell lines (HNSCCs) at 1:1 ratio in culture medium. Fibroblasts or HNSCCs cultured alone were used as controls. Similar co-cultures were set up between primary human macrophages and HNSCCs. Briefly, the culture medium was removed from differentiated macrophages and 1.5 × 10^5^ SCC099 or SCC154 cells were added in culture medium. Macrophages or HNSCCs cultured alone were used as controls. Co-cultures were incubated for 48 h prior to analysis of PD-1 ligand expression by flow cytometry. In addition to direct co-cultures, cells (HNSCC, fibroblasts, or macrophages) were treated with conditioned media and PD-L1/PD-L2 expression was assessed after 48 h by flow cytometry. Conditioned media were prepared from supernatants of confluent cultures of HNSCCs, fibroblasts and macrophages filtered through 0.2 μm pore membranes to remove viable and apoptotic cells. Where indicated, cells were cultured in the presence of ODN TTAGGG (3.9 μM, InvivoGen; #tlrl-ttag151), chloroquine (10 μM, InvivoGen; #tlrl-chq) or neutralizing antibodies against interferon-γ (IFN-γ, 5 μg/ml, R&D Systems; MAB285), tumor necrosis factor-α (TNF-α, 5 μg/ml, R&D Systems; #MAB610) and CD81/TAPA-1 (5 μg/ml, Abcam; #ab35026).

### Cell Line Authentication

UPCI-SCC-099 (SCC099) and UPCI-SCC-154 (SCC154) HNSCC cell lines were received directly from DSMZ (Braunschweig, Germany), which used short tandem repeat (STR) DNA profiling for their authentication. Primary BJ human fibroblasts were obtained from ATCC, which used STR profiling for their characterization. SCCs and fibroblasts were passaged for <3 months and therefore re-authentication was not required.

### Immunohistochemistry

The following primary antibodies were used: mouse monoclonal CD4 (#NCL-L-CD4-368); mouse monoclonal CD8 (#NCL-L-CD8-4B11); mouse monoclonal CD68 (#NCL-L-CD68) and mouse monoclonal CD163 (#NCL-L-CD163) (all from Novocastra, Leica Biosystems); rabbit polyclonal anti-inducible nitric oxide synthase (iNOS, Abcam; #ab3523); goat polyclonal PD-1 (#AF1086); and goat polyclonal PD-L2 (#AF1224) (both R&D Systems); and mouse monoclonal CD274 (PD-L1) (Biolegend; #329702). Paraffin sections of tumor tissue were cut at 4 μm and heated for 45 min at 60°C prior to staining. Heat antigen retrieval was carried out using Epitope Retrieval Solution 1 (for all antibodies except CD68), pH 6 or Epitope Retrieval Solution 2 (CD68), pH 9 at 100°C for 20 or 30 min, according to the antibody. Antibodies were diluted 1:50 (CD8, PD-1, PD-L1) or 1:100 for the remaining antibodies, and incubated for 15 min. Negative controls used antibody diluent in place of primary antibody. Visualization for mouse monoclonal antibodies was carried out using the Bond Polymer Refine Detection kit, an HRP-conjugated 3,3'-diaminobenzidine (DAB) detection system (Leica Biosystems; #DS9800). Visualization for goat polyclonal antibodies was by Bond Intense R kit, a Biotin/streptavidin HRP-conjugated DAB detection system, supplied by Leica Microsystems and secondary antibody biotinylated rabbit anti-goat (Dako; #E0466). All immunohistochemistry staining was carried out using Bond III Fully automated staining system and associated reagents, supplied by Leica Microsystems. Images were captured with a Hamamatsu Nanozoomer RS2.0 slide scanner, equipped with an Olympus objective (20 × and 40 × magnification) using the NDP.scan software. Images were generated using the NDP.view 2 software.

### Flow Cytometry Analysis

For detection of PD-L1 and PD-L2, fibroblasts and HNSCC cell lines (HNSCCs) cultured alone or in co-culture were detached by incubation with Trypsin/EDTA (Sigma-Aldrich) at 37°C, followed by washes in culture medium. Macrophages and HNSCCs cultured alone or in co-culture were collected by incubation in PBS containing 5 mM EDTA and 0.2% w/v bovine serum albumin for 15 min on ice and gentle scraping. Cells were washed several times in PBS with 2% FCS and stained for surface expression of epithelial cell adhesion molecule (EpCAM) (APC; R&D Systems; #FAB9601A) to distinguish HNSCCs from fibroblasts and macrophages. Additionally, cells were stained with PE-conjugated monoclonal antibodies against PD-L1 (#12-5983-42) and PD-L2 (#12-5888-42) (both eBioscience) or PE-conjugated isotype control antibodies (BD Biosciences; #555749). Initial experiments for detection of PD-L1 expression used a mouse anti-human-PD-L1 antibody (Biolegend; #329702) followed by staining with PE-conjugated goat anti-mouse antibody (Sigma-Aldrich; P9287). For macrophage-HNSCCs co-cultures, 7-AAD (BD Biosciences; #559925) was used to exclude dead cells from the analysis. Samples were acquired on a FACSCalibur (BD Biosciences) flow cytometer and data analysis was performed using FlowJo software version 7. Mean fluorescence intensity (MFI) was calculated by subtracting the MFI of samples stained with PE-conjugated isotype control from the MFI of samples stained with PD-L1 and PD-L2, respectively. MFI for samples indirectly stained with PD-L1/GAM-PE was adjusted to match MFI of samples stained with PE-conjugated PD-L1.

### Quantification of Cytokines in Culture Supernatants

Supernatants from co-cultures of fibroblasts and HPV-positive HNSCCs were stored frozen until quantification of IFN-γ and TNF-α by DuoSet ELISA (R&D Systems; #DY285, IFN-γ; #DY210, TNF-α).

### Statistical Analysis

One-way ANOVA with Bonferroni post-test for multiple comparisons and unpaired two-tailed Student's *t*-test were performed using GraphPad Prism assuming independent samples and normal distribution (as indicated in the Figure legends). Probability (*p*) values of < 0.05 were considered statistically significant.

## Results

### PD-L1 and PD-L2 Are Expressed Both in Tumor Cells and Stroma in HNSCC Tissue

**Figure 1:**
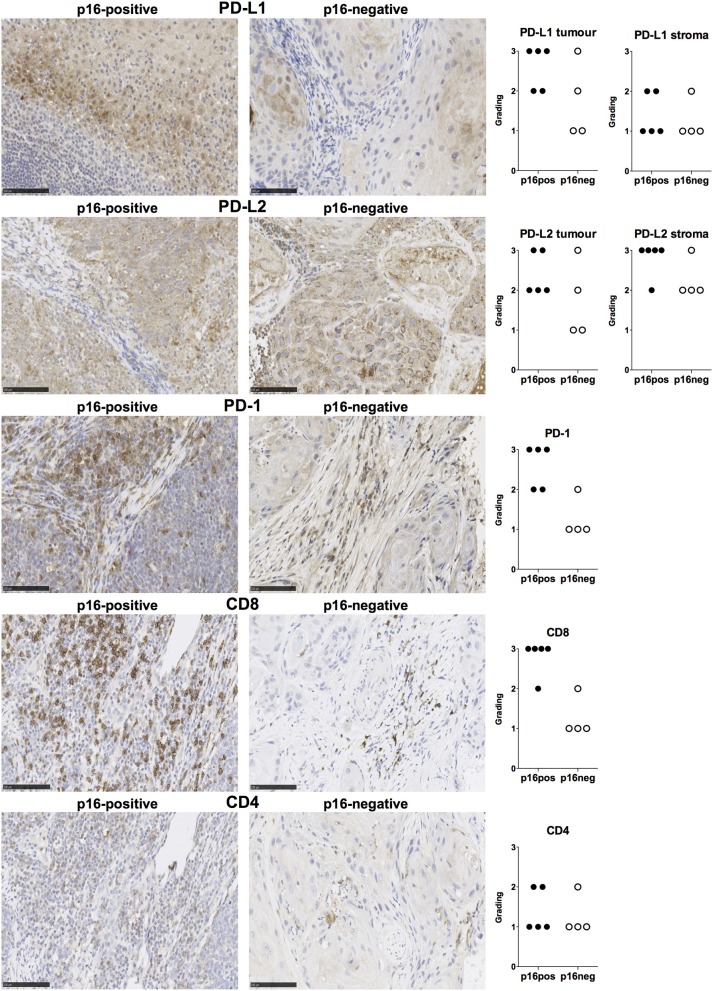
PD-L1, PD-L2, PD-1, CD8, and CD4 expression in p16-positive and p16-negative HNSCC. PD-L1, PD-L2, PD-1, CD8, and CD4 expression was assessed in tumor biopsy tissue from five p16-positive and four p16-negative HNSCC patients using immuno-histochemistry (details in Methods). Representative staining (scale bars, 100 μm) and cumulative data of marker expression (grading scale PD-L1/PD-L2: 1, low; 2, moderate; 3 high expression; grading scale PD-1, CD8, CD4: 1, <50 cells/field; 2, 50–150 cells/field; 3, >150 cells/field).

**Figure 2:**
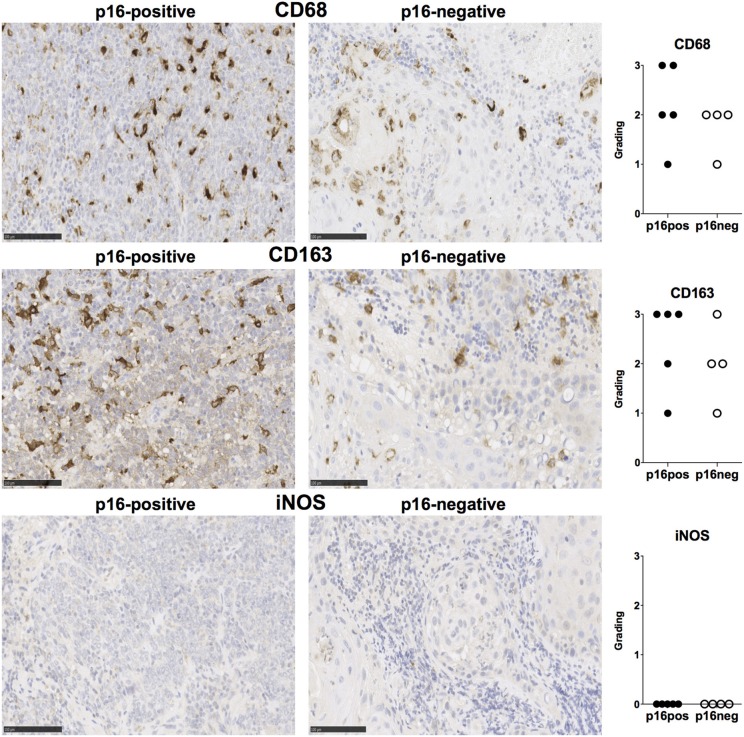
CD68, CD163, and iNOS expression in p16-positive and p16-negative HNSCC. Macrophage marker expression was assessed in tumor biopsy tissue from five p16-positive and four p16-negative HNSCC patients using immuno-histochemistry. Representative staining (scale bars, 100 μm) and cumulative data of marker expression (grading scale: 1, <50 cells/field; 2, 50–100 cells/field; 3, >100 cells/field).

We examined expression of PD-L1 and PD-L2 in nine treatment-naïve HNSCC tumor tissue samples using immunohistochemistry (clinical details in Table 1). PD-L1 was expressed in all tissue samples and was present both in tumor cells and stromal tissue (Figure 1). Similarly, PD-L2 was also present in all tissue samples examined and both tumor cells and stroma expressed PD-L2 *in situ* (Figure 1). Semi-quantitative analysis did not reveal differences in the expression of PD-1 ligands in p16-positive and p16-negative HNSCC tissue (Figure 1). We next examined PD-1 expression in the HNSCC tissue. An infiltrate of PD-1^+^ cells was noted in all HNSCC samples (Figure 1). Moreover, all HNSCC samples also exhibited CD8^+^ cell infiltration (Figure 1). Fewer PD-1^+^ and CD8^+^ cells were present in p16-negative samples (Figure 1). Compared to CD8 expression, relatively lower numbers of CD4^+^ cells were found in HNSCC tumor tissue (Figure 1). We also examined macrophage presence in HNSCC tumor tissue using CD68 (pan-macrophage marker), iNOS (M1 marker) and CD163 (M2 marker). CD68^+^ and CD163^+^ macrophages were noted in all HNSCC, while iNOS-positive macrophages were not identified (Figure 2).

### HPV-Positive HNSCCs Up-Regulate PD-L1 and PD-L2 Expression on Fibroblasts

**Figure 3:**
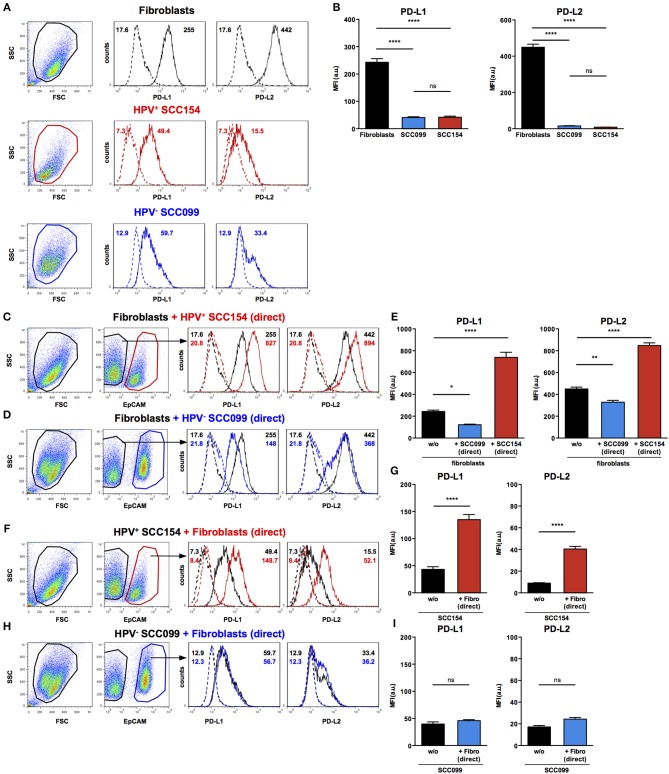
HPV-positive HNSCCs increase PD-L1 and PD-L2 on fibroblasts. PD-L1 and PD-L2 expression on primary BJ human fibroblasts, HPV-positive (SCC154), and HPV-negative (SCC099) HNSCC cell lines (HNSCCs) was detected by flow cytometry. Illustrative histograms show PD-L1 and PD-L2 expression on fibroblasts (black histograms), HPV-positive (red histograms), or HPV-negative (blue histograms) HNSCCs **(A)**. Graphs show PD-L1 and PD-L2 expression (mean ± SEM; *n* = 5) in fibroblasts, HPV-positive, and HPV-negative HNSCCs **(B)**. Fibroblasts were cultured alone or co-cultured in direct contact (direct) with HPV-positive (SCC154) or HPV-negative (SCC099) HNSCCs. Fibroblasts were identified in co-cultures by lack of EpCAM expression. Illustrative histograms show PD-L1 and PD-L2 expression on fibroblasts cultured alone (black histograms) or co-cultured directly with HPV-positive SCC154 (**C**; red histograms) or HPV-negative SCC099 (**D**; blue histograms). Graphs show PD-L1 and PD-L2 expression (mean ± SEM; *n* = 5) on fibroblasts cultured alone (w/o) or co-cultured directly with HNSCC cells **(E)**. HPV-positive (SCC154) or HPV-negative (SCC099) HNSCCs were cultured alone or co-cultured in direct contact (direct) with fibroblasts. HNSCCs were identified in co-cultures by EpCAM expression. Illustrative histograms show PD-L1 and PD-L2 expression on HPV-positive SCC154 cultured alone (black histograms) or co-cultured directly with fibroblasts (**F**; red histograms). Graphs show PD-L1 and PD-L2 expression (mean ± SEM; *n* = 4) on HPV-positive SCC154 cultured alone or co-cultured directly with fibroblasts **(G)**. Illustrative histograms show PD-L1 and PD-L2 expression on HPV-negative SCC099 cultured alone (black histograms) or co-cultured directly with fibroblasts (**H**; blue histograms). Graphs show PD-L1 and PD-L2 expression (mean ± SEM; *n* = 4) on HPV-negative SCC099 cultured alone or co-cultured directly with fibroblasts **(I)**. *p < 0.05; **p < 0.01; ****p < 0.0001; ns, not significant (one-way ANOVA with Bonferroni correction for multiple comparisons) Numbers adjacent to plots represent MFI values; dashed histograms show control staining with isotype-matched antibodies. MFI, mean fluorescence intensity.

As we found that PD-1 ligands were expressed in both tumor and stroma of HNSCC tissues, we examined if interaction between HNSCC tumor cells and fibroblasts could influence expression of PD-1 ligands. We therefore, tested the effect of HPV-positive (SCC154) and HPV-negative (SCC099) HNSCC cell lines (HNSCCs) on PD-1 ligand expression on BJ fibroblasts in an *in vitro* co-culture system. Fibroblasts expressed high baseline levels of PD-L1 and PD-L2, whilst HPV-positive and HPV-negative HNSCCs expressed low levels (Figures 3A,B). Interestingly, expression of PD-L1 on fibroblasts increased significantly upon co-culture with HPV-positive HNSCCs (Figures 3C,E, *p* < 0.0001). In contrast, HPV-negative HNSCCs decreased PD-L1 on fibroblasts (Figures 3D,E). The same pattern was noted for PD-L2 expression on fibroblasts: HPV-positive HNSCCs significantly increased PD-L2 levels (Figures 3C,E, *p* < 0.0001), whilst HPV-negative HNSCCs decreased PD-L2 (Figures 3D,E).

### Fibroblasts Up-Regulate PD-L1 and PD-L2 Expression in HPV-Positive HNSCCs

As HPV-positive HNSCCs up-regulated PD-1 ligands on BJ fibroblasts, we also examined whether fibroblasts had a reciprocal effect on PD-1 ligands expression on HNSCCs. Interaction with fibroblasts in co-culture significantly increased PD-L1 and PD-L2 expression on HPV-positive HNSCCs (Figures 3F,G, *p* < 0.0001). In contrast, PD-L1 and PD-L2 expression on HPV-negative HNSCCs did not change following co-culture with fibroblasts (Figures 3H,I).

### Macrophages Up-Regulate PD-L1 and PD-L2 on HPV-Positive HNSCCs

**Figure 4:**
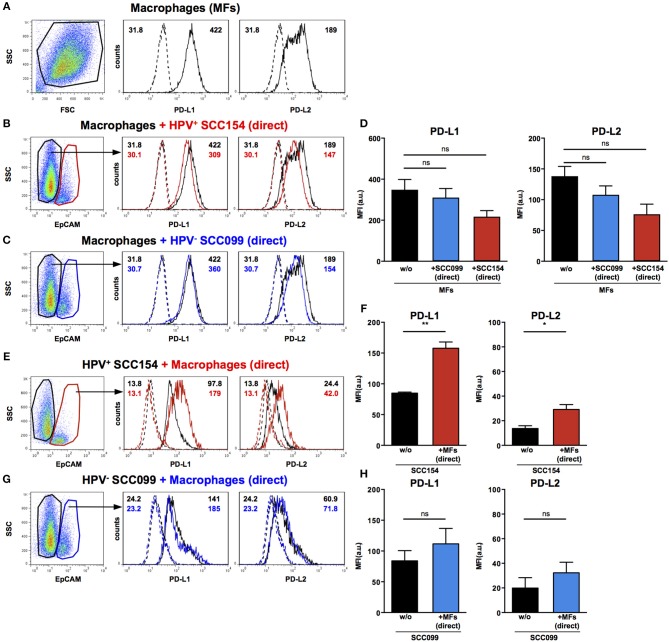
PD-L1 and PD-L2 expression in co-cultures of macrophages and HPV-positive or HPV-negative HNSCCs. Macrophages were cultured alone (w/o) or co-cultured in direct contact (direct) with HPV-positive (SCC154) or HPV-negative (SCC099) HNSCC cell lines (HNSCCs). Illustrative histograms show PD-L1 and PD-L2 expression on macrophages cultured alone (**A**; black histograms). Macrophages were identified in co-cultures by lack of EpCAM expression. Illustrative histograms show PD-L1 and PD-L2 expression on macrophages cultured alone (black histograms) or co-cultured directly with HPV-positive SCC154 (**B**; red histograms) or HPV-negative SCC099 (**C**; blue histograms). Graphs show PD-L1 and PD-L2 expression (mean ± SEM; *n* = 4) on macrophages cultured alone (w/o) or co-cultured directly with HNSCCs **(D)**. HPV-positive (SCC154) or HPV-negative (SCC099) HNSCCs were cultured alone or co-cultured in direct contact (direct) with macrophages. HNSCCs were identified in co-cultures by EpCAM expression. Illustrative histograms show PD-L1 and PD-L2 expression on HPV-positive SCC154 cultured alone (black histograms) or co-cultured directly with macrophages (**E**; red histograms). Graphs show PD-L1 and PD-L2 expression (mean ± SEM; *n* = 3) on HPV-positive SCC154 cultured alone or co-cultured directly with macrophages **(F)**. Illustrative histograms show PD-L1 and PD-L2 expression on HPV-negative SCC099 cultured alone (black histograms) or co-cultured directly with macrophages (**G**; blue histograms). Graphs show PD-L1 and PD-L2 expression (mean ± SEM; *n* = 3) on HPV-negative SCC099 cultured alone or co-cultured directly with macrophages **(H)**. *p < 0.05; **p < 0.01; ns, not significant (**D**: one-way ANOVA with Bonferroni correction for multiple comparisons; **F,H**: unpaired two-tailed Student's *t*-test) Numbers adjacent to plots represent MFI values; dashed histograms show control staining with isotype-matched antibodies. MFI, mean fluorescence intensity.

Macrophages are another important constituent of tumor stroma and therefore, we tested the effect of HNSCCs on PD-1 ligand expression by macrophages. Primary peripheral blood monocyte-derived human macrophages expressed both PD-L1 and PD-L2 (Figure 4A). In contrast to our findings on fibroblasts, co-culture with HPV-positive HNSCCs did not increase PD-L1 and PD-L2 expression on macrophages (Figures 4B,D). Similarly, HPV-negative HNSCCs did not affect PD-1 ligands expression on macrophages (Figures 4C,D). We also examined the effect of macrophages on PD-1 ligand expression by HNSCCs. PD-L1 and PD-L2 expression on HPV-positive HNSCCs increased significantly following direct co-culture with macrophages (Figures 4E,F), although the effect was more marked for PD-L1 than for PD-L2. In contrast, macrophages did not alter PD-1 ligands expression on HPV-negative HNSCCs (Figures 4G,H).

### Conditioned Medium From HPV-Positive HNSCCs Up-Regulates PD-L1 and PD-L2 on Fibroblasts

**Figure 5:**
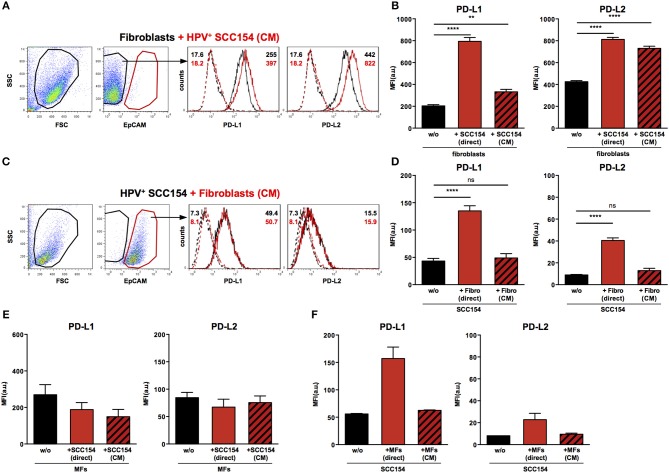
Conditioned medium from HPV-positive HNSCCs up-regulates PD-L1 and PD-L2 on fibroblasts. Fibroblasts were cultured alone or co-cultured in direct contact with HPV-positive SCC154 (direct) or with conditioned medium from HPV-positive SCC154 (CM). Illustrative histograms show PD-L1 and PD-L2 expression on fibroblasts cultured alone (black histograms) or co-cultured with conditioned medium from HPV-positive SCC154 (**A**; red histograms). Graphs show PD-L1 and PD-L2 expression (mean ± SEM; *n* = 13) on fibroblasts cultured alone (w/o), co-cultured directly with HPV-positive SCC154 (direct) or with conditioned medium from HPV-positive SCC154 (CM) **(B)**. HPV-positive (SCC154) HNSCCs were cultured alone or co-cultured in direct contact with fibroblasts (Fibro direct) or with conditioned medium from fibroblasts (Fibro CM). Illustrative histograms show PD-L1 and PD-L2 expression on HPV-positive SCC154 cultured alone (black histograms) or co-cultured with conditioned medium from fibroblasts (**C**; red histograms). Graphs show PD-L1 and PD-L2 expression (mean ± SEM; *n* = 4) on HPV-positive SCC154 cultured alone (w/o), co-cultured directly with fibroblasts (Fibro direct) or with conditioned medium from fibroblasts (Fibro CM) **(D)**. Macrophages were cultured alone (w/o) or co-cultured in direct contact with HPV-positive SCC154 (direct) or with conditioned medium from HPV-positive SCC154 (CM). Graphs show PD-L1 and PD-L2 expression (mean ± SEM; *n* = 3) on macrophages for the indicated conditions **(E)**. HPV-positive SCC154 HNSCCs were cultured alone (w/o) or co-cultured in direct contact with macrophages (MFs direct) or with conditioned medium from macrophages (MFs CM). Graphs show PD-L1 and PD-L2 expression (mean ± SEM; *n* = 2) on HPV-positive SCC154 HNSCCs for the indicated conditions **(F)**. **p < 0.01; ****p < 0.0001; ns, not significant (one-way ANOVA with Bonferroni correction for multiple comparisons) Numbers adjacent to plots represent MFI values; dashed histograms show control staining with isotype-matched antibodies. MFI = mean fluorescence intensity.

We next analyzed the mechanisms underlying the up-regulation of PD-1 ligands on fibroblasts upon co-culture with HPV-positive HNSCCs. To determine whether this was contact dependent or mediated via soluble factors, fibroblasts were cultured either with HPV-positive HNSCCs or with conditioned medium (supernatant) from these cells (details in Methods). Treatment with conditioned medium induced significant PD-L1 up-regulation on fibroblasts (Figures 5A,B). However, the effect of conditioned medium was smaller than that observed with direct co-culture: whilst direct co-culture with HPV-positive HNSCCs resulted in four-fold up-regulation of PD-L1, conditioned medium induced a 1.6 fold increase (Figures 5A,B). Next we analyzed the effect of conditioned medium on PD-L2 expression. Significant up-regulation of PD-L2 was observed on fibroblasts cultured with conditioned medium from HPV-positive HNSCCs (Figures 5A,B). Of note, the effect of conditioned medium on PD-L2 expression on fibroblasts was similar to that induced by direct co-culture with HPV-positive HNSCCs. As fibroblasts increased PD-1 ligand expression on HPV-positive HNSCCs in co-culture (Figures 3F,G), we tested whether this was also the case with conditioned medium from fibroblasts. Conditioned medium from cultures of fibroblasts did not alter PD-L1 and PD-L2 ligand expression on HPV-positive HNSCCs (Figures 5C,D). We also assessed the effect of HPV-positive HNSCCs conditioned medium on macrophages and found no change on PD-L1 and PD-L2 expression (Figure 5E). Similarly, conditioned medium from macrophages had no effect on PD-L1 and PD-L2 expression on HPV-positive HNSCCs (Figure 5F).

### The Up-Regulation of PD-1 Ligands on Fibroblasts Induced by HPV-Positive HNSCCs Is Not Mediated by Interferon-γ (IFN-γ), Tumor Necrosis Factor-α (TNF-α), or Tetraspanin CD81

**Figure 6:**
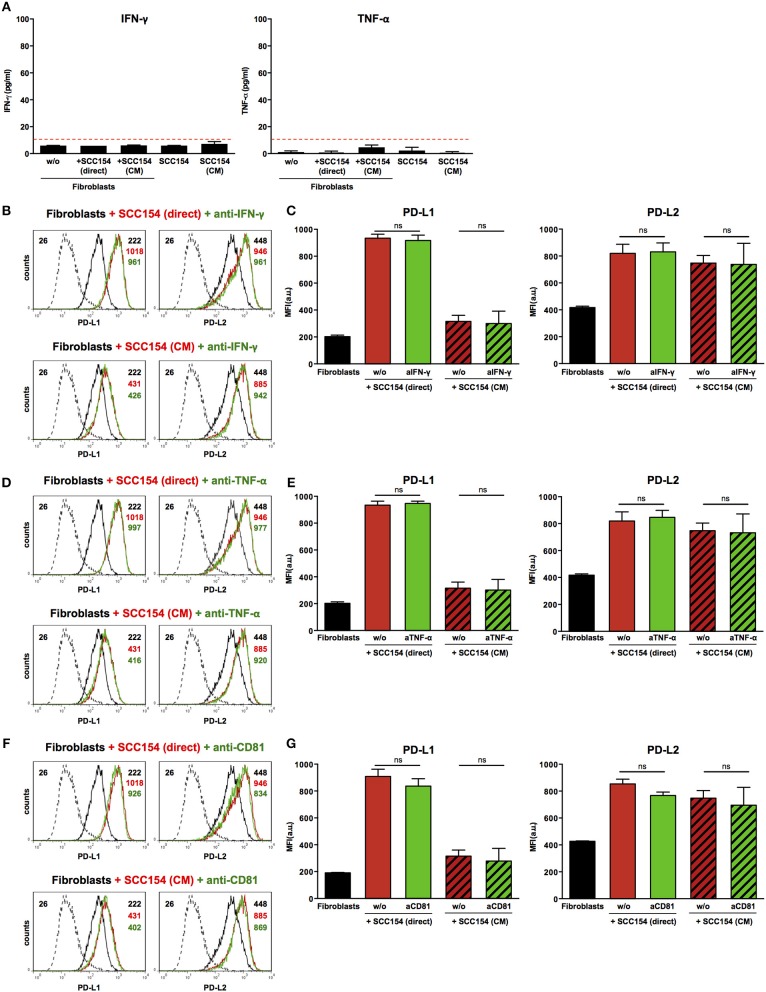
Blockade of IFN-γ, TNF-α, or CD81 does not affect PD-L1 and PD-L2 up-regulation by HPV-positive HNSCCs. Fibroblasts were cultured alone (w/o) or co-cultured in direct contact with HPV-positive SCC154 (SCC154 direct) or with conditioned medium from HPV-positive SCC154 (SCC154 CM) as indicated. Graphs **(A)** show IFN-γ and TNF-α levels in culture supernatants (mean ± SEM; *n* = 4). The dashed red line indicates the lowest value (15.6 pg/ml) of the dynamic range for the ELISA assays used. Neutralizing antibodies anti-IFN-γ **(B,C)**, anti-TNF-α **(D,E)**, or anti-CD81 **(F,G)** were added to the cultures as indicated. Illustrative histograms show PD-L1 and PD-L2 expression on fibroblasts cultured alone (black histograms), co-cultured directly with HPV-positive SCC154 or with conditioned medium from HPV-positive SCC154 alone (red histograms) or in the presence of blocking antibodies (green histograms). Graphs show PD-L1 and PD-L2 expression (mean ± SEM; *n* = 3) on fibroblasts for the indicated treatments. ns, not significant (one-way ANOVA with Bonferroni correction for multiple comparisons) Numbers adjacent to plots represent MFI values; dashed histograms show control staining with isotype-matched antibodies. MFI, mean fluorescence intensity.

Inflammatory cytokines such as IFN-γ have been proposed to mediate PD-L1 up-regulation on several cell types (19, 20). Moreover, tumor-infiltrating lymphocytes were proposed to induce PD-L1 expression in HNSCC via IFN-γ (23). We therefore tested supernatants from fibroblasts co-cultured with HPV-positive HNSCCs or with conditioned medium from these cells for inflammatory cytokines. No IFN-γ or TNF-α was present in supernatants from fibroblasts cultured alone or in the presence of HPV-positive HNSCCs or conditioned medium from these cells (Figure 6A). In addition, blocking IFN-γ or TNF-α with neutralizing antibodies did not have any impact on PD-L1 and PD-L2 up-regulation on fibroblasts cultured with HPV-positive HNSCCs or conditioned medium (Figures 6B–E). We also investigated whether transfer of PD-1 ligands via microvesicles derived from HPV-positive HNSCCs could account for PD-L1 and PD-L2 increase on fibroblasts. We therefore targeted tetraspanin CD81 (TAPA-1), which is a common component of tetraspanin-enriched microdomains in the membrane of microvesicles and has been implicated in the entry of viruses like HPV and hepatitis C virus in epithelial cells (34). Blocking antibodies against tetraspanin CD81 did not inhibit PD-L1 and PD-L2 up-regulation by fibroblasts co-cultured with HPV-positive HNSCCs or conditioned medium from these cells (Figures 6F,G).

### The Up-Regulation of PD-L1 and PD-L2 on Fibroblasts by HPV-Positive HNSCCs Is Mediated via Toll-Like Receptor-9 (TLR9)

**Figure 7:**
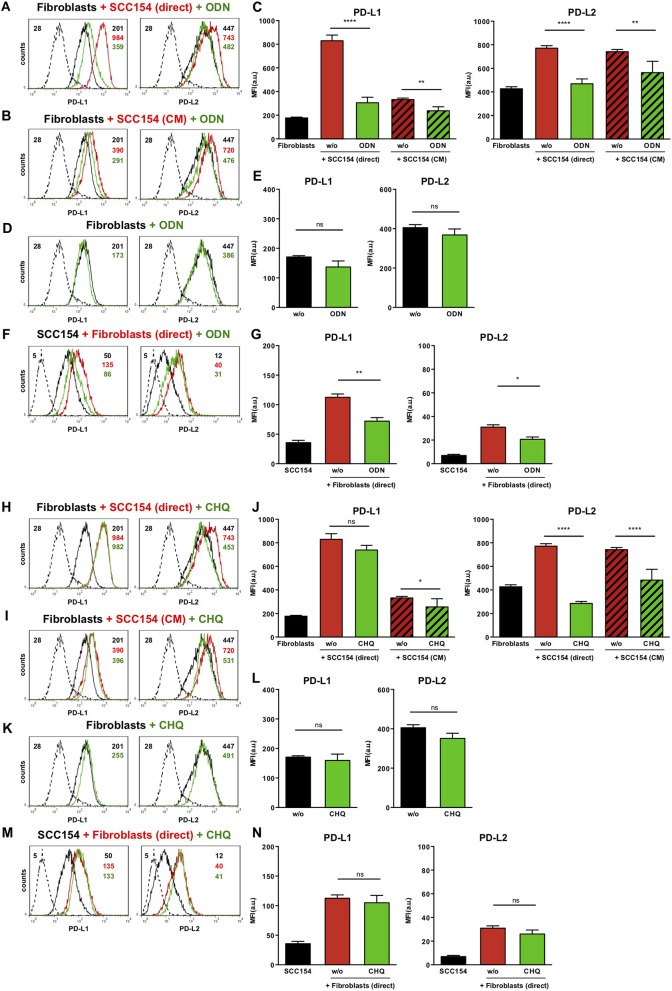
The TLR9 antagonists ODN TTAGGG and chloroquine inhibit PD-1 ligands up-regulation on fibroblasts co-cultured with HPV-positive HNSCCs. Fibroblasts were cultured alone (w/o) or co-cultured in direct contact with HPV-positive SCC154 (SCC154 direct) or with conditioned medium from HPV-positive SCC154 (SCC154 CM) in the presence or absence of the TLR9 antagonists ODN TTAGGG (ODN) or chloroquine (CHQ). Illustrative histograms show PD-L1 and PD-L2 expression on fibroblasts cultured alone (black histograms), co-cultured directly with HPV-positive SCC154 (red histograms) or co-cultured directly with HPV-positive SCC154 in the presence of ODN **(A)** or CHQ **(H)** (green histograms). Illustrative histograms show PD-L1 and PD-L2 expression on fibroblasts cultured alone (black histograms), cultured with conditioned medium from HPV-positive SCC154 (red histograms) or with conditioned medium from HPV-positive SCC154 in the presence of ODN **(B)** or CHQ **(I)** (green histograms). Graphs show PD-L1 and PD-L2 expression (mean ± SEM; *n* = 6) on fibroblasts for the indicated treatments **(C,J)**. Illustrative histograms show PD-L1 and PD-L2 expression on fibroblasts cultured alone (black histograms) or in the presence of ODN **(D)** or CHQ **(K)** (green histograms). Graphs show PD-L1 and PD-L2 expression (mean ± SEM; *n* = 4) on fibroblasts for the indicated treatments **(E,L)**. HPV-positive SCC154 were cultured alone or co-cultured in direct contact (direct) with fibroblasts in the presence or absence of TLR9 inhibitor ODN TTAGGG (ODN) or chloroquine (CHQ). Illustrative histograms show PD-L1 and PD-L2 expression on HPV-positive SCC154 cultured alone (black histograms), co-cultured directly with fibroblasts alone (red histograms), or co-cultured directly with fibroblasts in the presence of ODN **(F)** or CHQ **(M)** (green histograms). Graphs show PD-L1 and PD-L2 expression (mean ± SEM; *n* = 6) on HPV-positive SCC154 cultured alone or for the indicated co-culture conditions **(G,N)**. *p < 0.05; **p < 0.01; ****p < 0.0001; ns, not significant (one-way ANOVA with Bonferroni correction for multiple comparisons) Numbers adjacent to plots represent MFI values; dashed histograms show control staining with isotype-matched antibodies. MFI, mean fluorescence intensity.

Toll-like receptors (TLR) have been implicated in up-regulation of PD-L1 in antigen presenting cells (35). Endosomal TLR9 is a primary sensor for bacterial and viral DNA (36). As HPV is a double stranded DNA virus we next investigated whether TLR9 was involved in PD-L1 and PD-L2 expression by fibroblasts co-cultured with HPV-positive HNSCCs. For this purpose we used the oligonucleotide ODN TTAGGG (A151), which is a specific antagonist of human TLR9 (37, 38). Notably, ODN TTAGGG abrogated the up-regulation of both PD-L1 and PD-L2 in fibroblasts co-cultured with HPV-positive HNSCCs (Figures 7A,C), whilst it had no effect on the baseline expression of PD-1 ligands on fibroblasts (Figures 7D,E). Furthermore, ODN TTAGGG significantly inhibited the up-regulation of PD-L1 and PD-L2 in fibroblasts cultured with conditioned medium from HPV-positive HNSCCs (Figures 7B,C). As fibroblasts increased the expression of PD-1 ligands on HPV-positive HNSCCs (Figures 3F,G), we tested whether this effect was also mediated by TLR9. Indeed, ODN TTAGGG hindered the up-regulation of PD-L1 and PD-L2 on HPV-positive HNSCCs cultured with fibroblasts (Figures 7F,G). In addition to ODN TTAGGG, we tested another TLR9 inhibitor, chloroquine, which works by blocking endosomal acidification that is required for optimal TLR9 activation, and by inhibiting the binding of DNA to TLR9 (39, 40). As observed with ODN TTAGGG, chloroquine inhibited the up-regulation of PD-L2 by fibroblasts cultured either directly with HPV-positive HNSCCs or with conditioned medium from these cells (Figures 7H–J). In contrast to ODN TTAGGG, chloroquine did not affect PD-L1 expression on fibroblasts in these experiments (Figures 7H–J). Moreover, chloroquine had no effect on the baseline expression of PD-L1 and PD-L2 by fibroblasts (Figures 7K,L). In contrast to ODN TTAGGG, chloroquine also did not affect the fibroblast-induced up-regulation of PD-L1 and PD-L2 on HPV-positive HNSCCs (Figures 7M,N).

## Discussion

This work investigated the role of the inhibitory PD-1 pathway with focus on PD-L1 and PD-L2 in the stromal microenvironment in HNSCC. Here we show that PD-L1 and PD-L2 are expressed in both tumor cells and stroma *in situ* in treatment-naïve primary HSNCC. Of note, we demonstrate that HPV-positive but not HPV-negative HNSCC cell lines (HNSCCs) up-regulate both PD-L1 and PD-L2 expression on fibroblasts *in vitro* via a TLR9-depedent mechanism. To the best of our knowledge this is the first report identifying TLR9 as a new regulator of PD-1 ligand expression in fibroblasts in the context of tumor-stroma interaction. Our novel results suggest that HPV-positive HNSCC enlist fibroblasts and generate a tumor-permissive microenvironment via TLR9-mediated engagement of PD-1/PD-L1/PD-L2 pathways.

PD-L1 and PD-L2 are members of the B7 superfamily of co-stimulatory/co-inhibitory molecules (41) and bind to PD-1 expressed on T cells. In immune responses to tumors, PD-1 engagement on T cells is responsible for switching T lymphocytes off and preventing robust anti–tumor activity (4). Thereby PD-1 ligands have pivotal roles in maintaining an immunosuppressive microenvironment in tumors. In comparison to PD-L1, little information is available on PD-L2 expression in HNSCC. We show that both PD-1 ligands (PD-L1 and PD-L2) are expressed in HNSCC tissue *in situ* and are present in both tumor and stromal cells. Our findings on PD-L2 expression are in agreement with a recent study on tissue expression of PD-L2 from 40 HNSCC patients (13). This study also suggested that PD-L2 expression is a significant predictor of clinical response to pembrolizumab (an anti-PD-1 checkpoint inhibitor) and progression-free survival, independent of PD-L1 status in HNSCC (13). These data highlight a role for PD-L2 in HNSCC and suggest that PD-L2, in addition to PD-L1, has a role in tumor-stroma interactions in HNSCC *in vivo*. However, very little is known about the mechanisms regulating PD-L1 and in particular PD-L2 expression in HNSCC tumor and stroma.

We found constitutive expression of PD-L1 and PD-L2 in human primary BJ fibroblasts. Expression of PD-L2 has been previously described in fibroblasts from human intestinal mucosa (42) and from human lung cancers (43), although its role on fibroblasts is not completely understood. Notably, we found that co-culture with the HPV-positive HNSCC cell line (HNSCCs) significantly increased the expression of PD-L1 and PD-L2 on fibroblasts. This effect of HPV-positive HNSCCs was restricted to fibroblasts as it was not observed on primary human macrophages. Of note, fibroblasts and macrophages also increased the expression of PD-L1 and PD-L2 on HPV-positive HNSCCs. These results suggest a bi-directional interaction between HPV-positive tumor cells and stromal cells, resulting in an overall up-regulation of both PD-L1 and PD-L2.

A novel facet of our work unveils TLR9 as a receptor mediating the up-regulation of PD-L1 and PD-L2 on fibroblasts following interaction with HPV-positive HNSCCs. We found that ODN TTAGGG, a specific antagonist of TLR9, abrogated the up-regulation of both PD-L1 and PD-L2. Interestingly, chloroquine, another TLR9 inhibitor blocked PD-L2 up-regulation but had no effect on PD-L1. These findings may be explained by different mechanisms employed by ODN TTAGGG and chloroquine in TLR9 inhibition. TLR9 is an endosomal pattern recognition receptor for unmethylated 2'-deoxyribo(cytidine-phosphate-guanosine) (CpG) oligonucleotide (ODN) DNA motifs preferentially found in bacterial and viral DNA and rare in mammalian cells (36). ODN TTAGGG is believed to interfere with CpG ODN colocalisation with TLR9 in endosomes (44), whilst chloroquine hinders TLR9 activation by inhibiting endosomal acidification and blocking nucleic acid binding to TLR9 (39, 40). The different effects of ODN TTAGGG and chloroquine on PD-L1 and PD-L2 suggest further complexities in TLR9 regulation of PD-1 ligand expression that warrant investigation. In contrast to the effect on fibroblasts, HPV-positive HNSCCs did not up-regulate PD-L1 or PD-L2 on monocyte-derived macrophages. This may be due to low TLR9 expression in human monocyte-derived macrophages (45, 46).

Our *in vitro* results show a clear influence of HPV status on the expression of PD-L1 and PD-L2 by fibroblasts coming into contact with HNSCC cell lines. This is in keeping with *in situ* findings by Lyford-Pike et al. that PD-L1 expression was more common in HPV-positive HNSCC tissue (70%, *n* = 14/20 of their cohort) compared to HPV-negative samples (29%, *n* = 2/10) (23). In our study, immunohistochemistry staining did not reveal differences in PD-L1 and PD-L2 expression in treatment-naïve p16-positive compared to p16-negative HNSCC tissue samples (Figure 1), which may be due to the smaller sample size. Of note, a recent RNASeq gene expression profiling in more than 500 treatment-naïve HNSCC tissue samples did not identify differences in PD-L1 expression in HPV-positive and HPV-negative HNSCC, even though IFN-γ (an inducer of PD-L1) was significantly higher in the HPV-positive group (47). It is possible that in the tumor microenvironment, up-regulation of PD-1 ligands is influenced by multiple factors in addition to HPV. Quantification of PD-1 ligands solely in fibroblasts in tumor tissue *in situ* presents major challenges as no specific markers are available that can reliably identify fibroblasts *in situ*.

A limitation of our study is the use of an *in vitro* co-culture system to dissect the effect of the interactions between HNSCC cell lines and stromal cells on the expression of PD-1 ligands. For ethical and feasibility reasons, the fibroblasts used in this study were primary established cell lines and macrophages were generated from peripheral blood monocytes of normal donors. To investigate fibroblasts and macrophages directly isolated from HNSCC tumor tissue is challenging because of limited amounts of fresh tumor tissue available for research. Moreover, methods used to isolate macrophages and fibroblasts from tumor tissue and propagate these cells *in vitro* alter the phenotype and function of these cells.

Our results show that PD-1/PD-L1 and PD-1/PD-L2 pathways are important in the bidirectional interaction between tumor cells and stromal cells in HNSCC and could shape the anti-tumor immune response. We propose TLR9 as a new regulator of the interaction between HPV-positive HNSCCs and stromal cells, specifically fibroblasts, which controls PD-L1 and PD-L2 expression. Further dissection of TLR9's role in modulation of PD-1 ligands could reveal novel strategies to target PD-1 pathways and improve clinical outcomes in HPV-positive HNSCC.

## Data Availability

The generated datasets have been included in the manuscript and/or the supplementary material.

## Ethics Statement

This study was carried out in accordance with the recommendations of the Research Ethics Committee London-Chelsea who approved the study; all study subjects gave written informed consent in accordance with the Declaration of Helsinki.

## Author Contributions

PB designed and directed the study, recruited the patients, collected clinical data, designed experiments, analyzed data, and wrote the manuscript. ID designed and supervised the study, designed and performed experiments, analyzed data, and wrote the manuscript. JB designed and performed *in vitro* experiments, analyzed data, and edited the manuscript. PW analyzed data and provided critical discussion. ML provided access to patients and critical discussion. JK provided access to vital resources.

### Conflict of Interest Statement

The authors declare that the research was conducted in the absence of any commercial or financial relationships that could be construed as a potential conflict of interest.
